# Dephosphorylation of LjMPK6 by Phosphatase LjPP2C is Involved in Regulating Nodule Organogenesis in *Lotus japonicus*

**DOI:** 10.3390/ijms21155565

**Published:** 2020-08-03

**Authors:** Zhongyuan Yan, Jingjing Cao, Qiuling Fan, Hongmin Chao, Xiaomin Guan, Zhongming Zhang, Deqiang Duanmu

**Affiliations:** 1 State Key Laboratory of Agricultural Microbiology, College of Life Science and Technology, Huazhong Agricultural University, Wuhan 430070, China; yanzy@huas.edu.cn (Z.Y.); cjj123@webmail.hzau.edu.cn (J.C.); chaohongmin2014@163.com (H.C.); guximihubei@163.com (X.G.); zmzhang@mail.hzau.edu.cn (Z.Z.); 2 College of Life Science and Technology, Huazhong Agricultural University, Wuhan 430070, China; qlfan@mail.hzau.edu.cn

**Keywords:** LjPP2C, LjMPK6, *Lotus japonicus*, MAPK dephosphorylation, nodule development

## Abstract

The mitogen-activated protein kinase (MAPK) LjMPK6 is a phosphorylation target of SIP2, a MAPK kinase that interacts with SymRK (symbiosis receptor-like kinase) for regulation of legume-rhizobia symbiosis. Both LjMPK6 and SIP2 are required for nodulation in *Lotus japonicus*. However, the dephosphorylation of LjMPK6 and its regulatory components in nodule development remains unexplored. By yeast two-hybrid screening, we identified a type 2C protein phosphatase, LjPP2C, that specifically interacts with and dephosphorylates LjMPK6 in vitro. Physiological and biochemical assays further suggested that LjPP2C phosphatase is required for dephosphorylation of LjMPK6 in vivo and for fine-tuning nodule development after rhizobial inoculation. A non-phosphorylatable mutant variant LjMPK6 (T224A Y226F) could mimic LjPP2C functioning in MAPK dephosphorylation required for nodule development in hairy root transformed plants. Collectively, our study demonstrates that interaction with LjPP2C phosphatase is required for dephosphorylation of LjMPK6 to fine tune nodule development in *L. japonicus*.

## 1. Introduction

Root nodule symbiosis (RNS) between legumes and rhizobia allows conversion of atmospheric nitrogen into ammonia absorbed by plants. The establishment of RNS begins with mutualistic dialogue between two partners. Host plants secrete flavonoids, inducing rhizobia to synthesize and secrete Nod factors (NFs), a type of lipo-chitooligosaccharide molecules [1,2]. Two plant LysM-type serine/threonine receptor kinases, NFR1 and NFR5 in *Lotus japonicus* (Nod Factor Receptor 1 and 5) [3,4,5], cooperating with the leucine-rich repeat (LRR) receptor-like kinase SymRK (Symbiosis Receptor-like Kinase) [6], recognize NFs and initiate the NF signaling pathway [7,8,9,10]. The core component of the NF signaling pathway is a calcium oscillation which is decoded by the nucleus-localized CCaMK (calcium/calmodulin-dependent protein kinase) [11,12]. CCaMK phosphorylates and activates CYCLOPS [13,14], which binds to and activates the nodule inception (NIN) gene essential for rhizobial colonization, nodule organogenesis and ultimate nitrogen fixation in mature nodules [15,16].

Mitogen-activated protein kinase (MAPK) cascades play central roles in various intracellular signal transduction processes via sequential phosphorylation of three kinases, a MAPK kinase kinase (MAPKKK), a MAPK kinase (MAPKK) and a MAPK [17], culminating in phosphorylation of both threonine and tyrosine residues of the terminal MAPK components (e.g., MPK3/4/6) within their TXY consensus sequence [18,19]. Reversible phosphorylation and dephosphorylation affects MAPK protein structure and its activity toward downstream targets responsible for efficient cellular signal transduction. This process is achieved via two biochemical reactions performed by a specific pair of kinase and phosphatase, the latter being called MKP (MAPK phosphatase) [20]. Only a few MKP proteins have been identified so far. For example, the dual-specificity (DSP) phosphatase OsMKP1 dephosphorylates both phospho-threonine (pT) and phospho-tyrosine (pY) to negatively regulate the OsMKKK10-OsMKK4-OsMPK6 cascade regulating panicle architecture in rice [21,22]. Meanwhile, the type 2C protein phosphatase (PP2C)-type Ser/Thr phosphatase AP2C1 dephosphorylates pT in the ‘pTEpY’ loop of MPK3/6 to downregulate basal resistance and defense responses to *Pseudomonas syringae* in *Arabidopsis thaliana* [23].

The legume SIP2 is a MAPKK that directly interacts with SymRK. SIP2/SymRK interaction inhibits the kinase activity of SIP2 on the substrate LjMPK6 in *Lotus japonicu*s [24]. Both SIP2 and LjMPK6 are required for efficient nodulation [25]. However, the role of LjMPK6 dephosphorylation in RNS and its regulatory components remain elusive. Here, we showed that LjPP2C, a PP2C-type phosphatase, specifically interacts with and dephosphorylates LjMPK6 in vitro. Moreover, our molecular and phenotypic data suggest that LjPP2C contributes to the dephosphorylation of LjMPK6 in vivo, which is required for regulating nodule development in *L. japonicus*.

## 2. Results

### 2.1. LjPP2C Interacts with and Dephosphorylates LjMPK6 In Vitro

**Figure 1:**
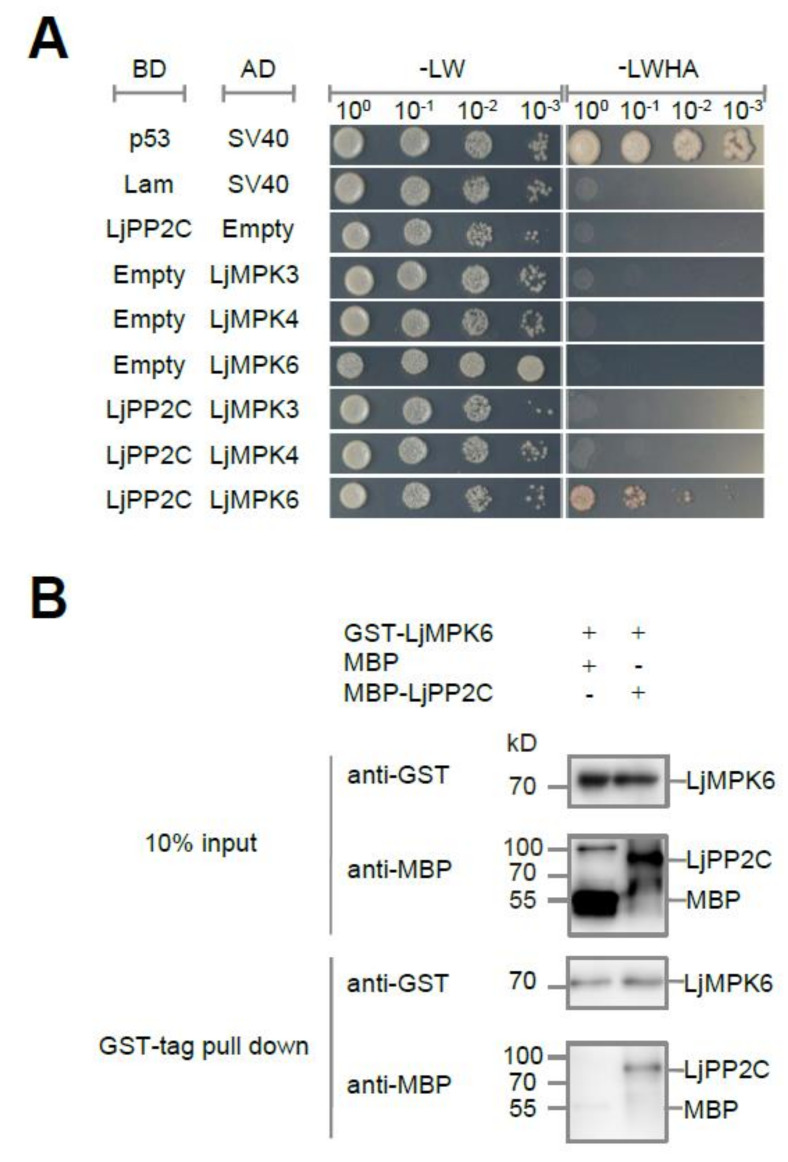
LjPP2C interacts with LjMPK6 in vitro. (**A**) Yeast two-hybrid assays shows interaction between LjPP2C and LjMPK6. Yeast cells were transformed with AD-LjMPK3/4/6 or BD-LjPP2C vectors. Serially diluted yeast cells were grown on SD-LW (lacking leucine and tryptophan) or SD-LWHA medium (lacking leucine, tryptophan, histidine and adenine). Interactions between p53/SV40 and Lam/SV40 were used as positive and negative controls, respectively. (**B**) GST pull-down of interaction between LjPP2C and LjMPK6. Positions of MBP-LjPP2C, MBP and GST-LjMPK6 are indicated. MBP-LiPP2C or MBP itself was incubated with GST-LjMPK6 and glutathione resin. After washing, retained proteins were analyzed by SDS-PAGE and immunoblotted with GST and MBP antibodies. +/-, with (+) or without (-) corresponding protein.

To study proteins interacting with the MAPK kinase LjMPK6, we identified several protein candidates from a Lotus cDNA library by yeast two-hybrid (Y2H) screening. One of these candidates is a type 2C protein phosphatase named LjPP2C (Lj2g3v2292680.1). The pairwise Y2H assay showed that LjPP2C interacted only with LjMPK6 and not with other MAP kinases such as LjMPK3 or LjMPK4, indicating that the interaction between LjPP2C and LjMPK6 is specific (Figure 1A). To further verify the direct physical interaction between two proteins, we purified recombinant MBP-tagged LjPP2C and GST-tagged LjMPK6 proteins from *E. coli* for in vitro pull-down assay. Our results confirmed that LjPP2C interacts with LjMPK6 directly (Figure 1B). 

**Figure 2:**
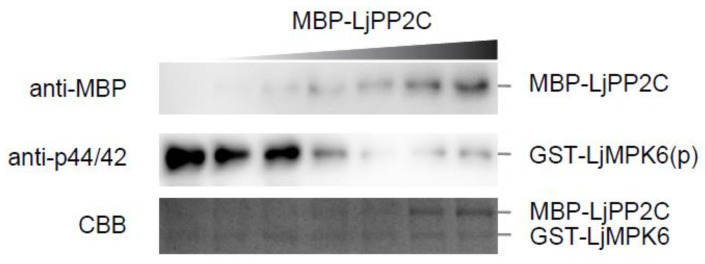
LjPP2C dephosphorylates LjMPK6 in vitro. Phosphorylation of LjMPK6 by SIP2 was performed in advance to prepare phosphorylated GST-tagged LjMPK6. An increasing amount of MBP-LjPP2C was then added to the reaction mixture. MBP-LjPP2C protein was detected by western blotting using anti-MBP antibody. Phosphorylated form of GST-LjMPK6 was detected by anti-p44/42 antibody. CBB, Coomassie Brilliant Blue staining.

To determine whether LjPP2C can use LjMPK6 as a dephosphorylation substrate, we performed in vitro phosphatase assay by incubating phosphorylated GST-LjMPK6 with MBP-LjPP2C for half an hour at room temperature. Immunoblot analysis using an antibody recognizing specifically the phosphorylated form of MPK6 showed that LjMPK6 phosphorylation level was reduced in a dosage-dependent manner with increasing amount of LjPP2C protein, corroborating the dephosphorylation activity of LjPP2C on LjMPK6 in vitro (Figure 2). 

### 2.2. Expression Pattern of LjPP2C in Both Non-Symbiotic and Symbiotic Tissues

**Figure 3:**
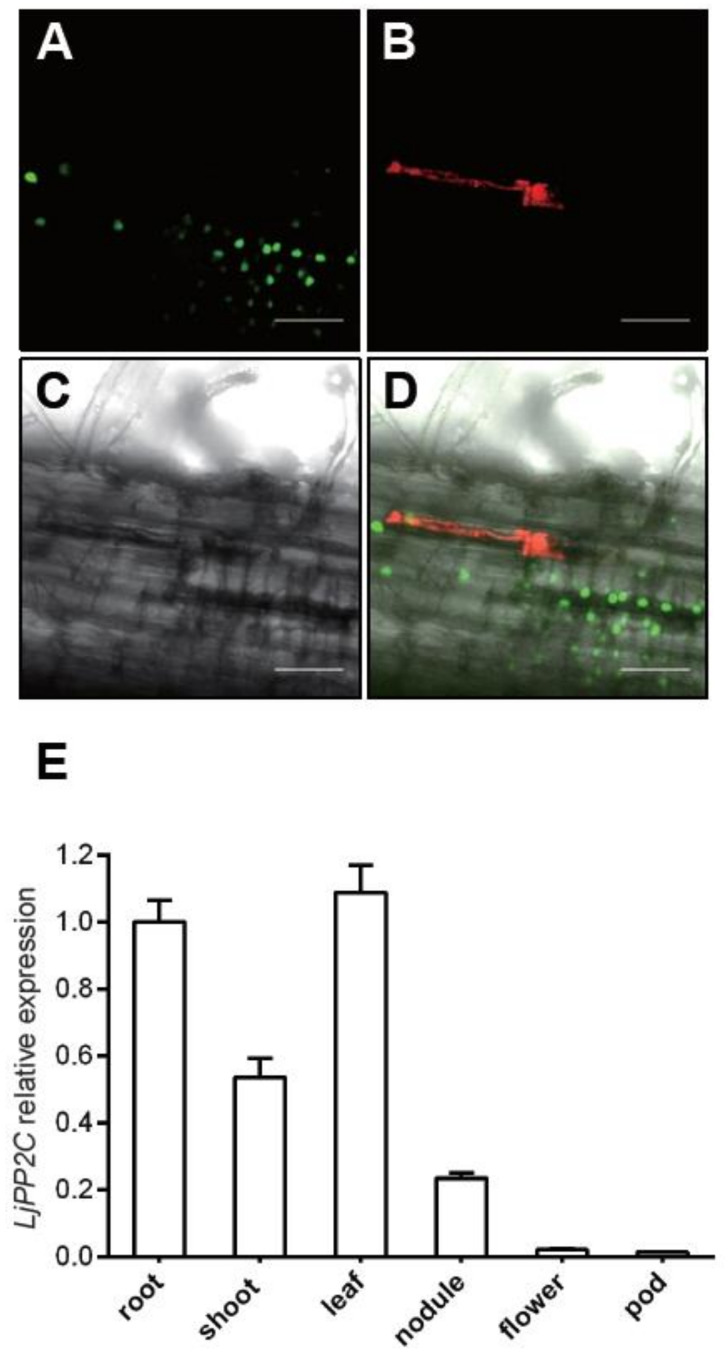
Analysis of *LjPP2C* expression pattern. (**A**–**D**) Images of *LjPP2C* promoter analysis in nodule primordium stage. MG20 plants were transformed with pUB-Hyg-LjPP2Cpro::tYFPnls construct. Positive hairy roots were inoculated with *M. loti* MAFF303099 (expressing mCherry). Transformed roots were visualized by GFP fluorescence (**A**), mCherry fluorescence (**B**), Brightfield (**C**) and Merge (**D**). Bars: 50 µm (**A**–**D**). (**E**) Expression of *LjPP2C* in different tissues. Roots were harvested at 14 days post inoculation (pdi) with MAFF303099. Nodules were harvested at 21 dpi. Total RNA was isolated and used for real-time PCR to quantify the expression levels of *LjPP2C* mRNA. The ATPase gene (AW719841) was used as the internal control. Error bars indicate SE of three technical replicates.

To investigate the expression pattern of *LjPP2C* in nodules, we constructed a fusion reporter gene containing the *LjPP2C* promoter and tYFPnls, which carries a nuclear localization signal, and introduced the plasmid into wild-type *L. japonicus* MG20 by stable transformation. We selected several positive transgenic plants for analysis and results from one representative plant was shown. Fluorescence microscopy analysis showed that the *LjPP2C* promoter was actively expressed in root cortex cells (Figure 3A-D). In addition, our qRT-PCR results showed that the relative expression level of *LjPP2C* is also higher in non-symbiotic tissues such as root, shoot and leaf, but to a much lower extent in flower and pod (Figure 3E). Overall, these results suggest that *LjPP2C* is not specifically expressed in symbiotic tissues.

### 2.3. LjPP2C is Required for Nodule Development 

**Figure 4:**
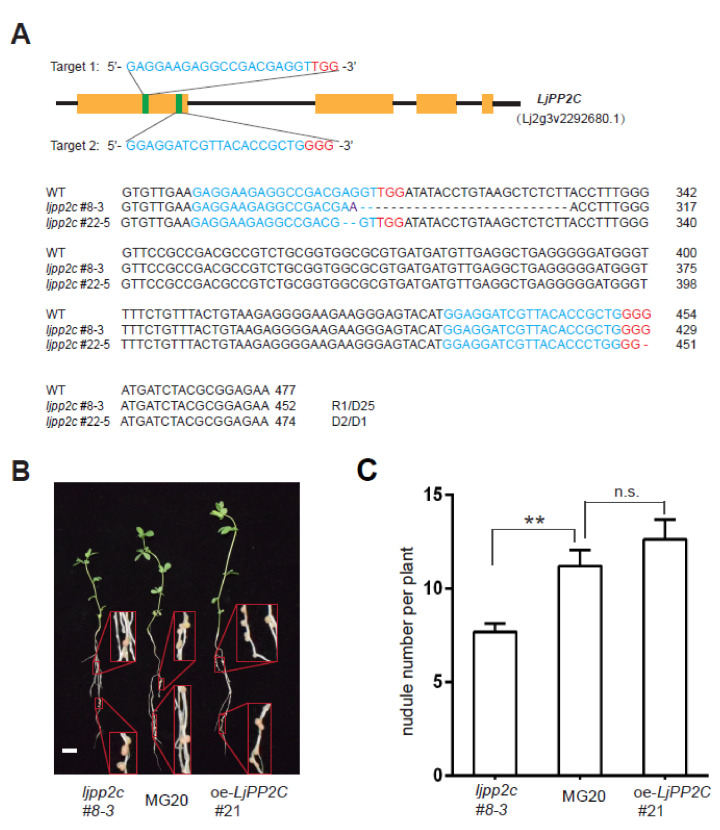
*LjPP2C* mutation affects nodule formation. (**A**) CRISPR/Cas9-mediated *LjPP2C* knockout in stable transgenic MG20 plants. Above: Genomic DNA structure of the *LjPP2C* gene. The PAM sequence of the *LjPP2C* sgRNA target is colored in red and the sgRNA target is in blue. Below: Indel mutations of two stable lines. Both lines have mutations leading to early translational termination. WT, wide-type control. D1/2/25, 1 bp/2 bp/25 bp DNA deletion; R1, 1 bp replacement. (**B**,**C**) Growth phenotype (**B**) and nodule numbers (**C**) of *ljpp2c*#8-3, MG20 and oe-*LjPP2C*#21 at 4 weeks post-inoculation (4 wpi). Bar in (**B**), 1 cm. Error bars in (**C**) indicate SE of 27~29 plants analyzed for each genotype. Student’s *t*-test was used for statistical comparisons. ** *p* < 0.01, n.s., not significant.

To further explore the regulatory role of *LjPP2C* in nodulation, we introduced a CRISPR/Cas9-mediated *LjPP2C* gene knockout construct into Lotus using a stable transformation procedure [26]. In parallel, to generate stable overexpression transgenic plants, we also introduced a *LjPP2C* overexpression construct in a similar way. Two independent knockout mutants of *LjPP2C* (*ljpp2c*#8-3, *ljpp2c*#22-5) were identified by PCR amplification of the target allele and DNA sequencing (Figure 4A).

We next performed nodulation assays under nitrogen-deficient conditions. The *ljpp2c*#8-3 mutant produced fewer nodules than wild-type MG20, whereas *LjPP2C* overexpression line (oe-*LjPP2C*#21) showed marginally, but insignificantly increased nodule numbers, indicating that *LjPP2C* plays an important role in nodule formation (Figure 4B,C).

### 2.4. LjPP2C is Required for MAPK Dephosphorylation In Vivo

**Figure 5:**
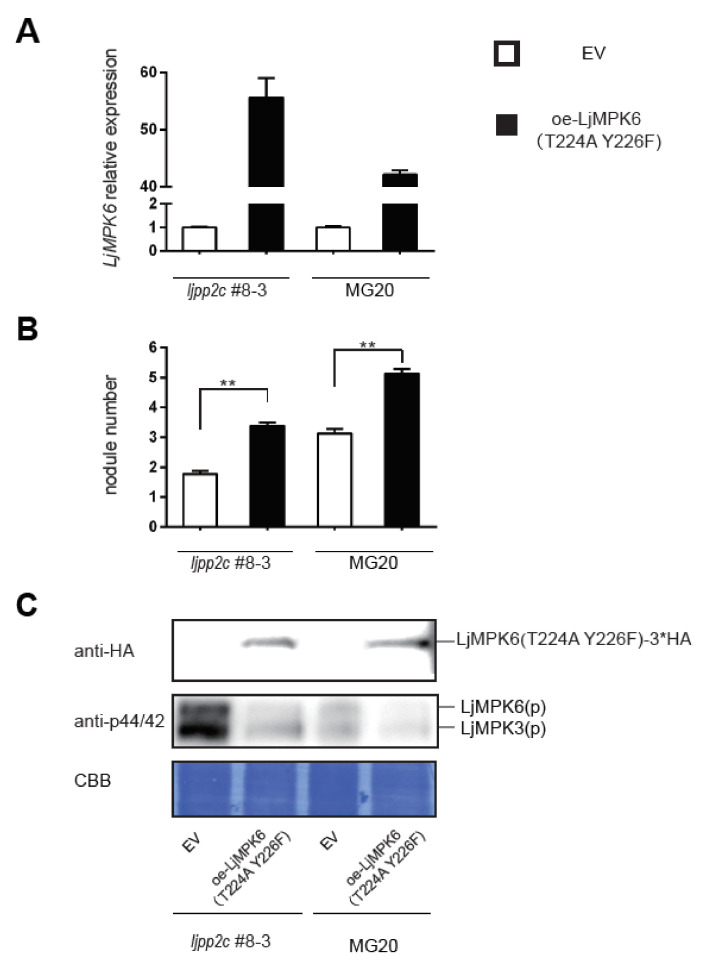
Overexpression of non-phosphorylatable LjMPK6 increased nodule formation in WT and *ljpp2c* mutant plants. MG20 and *ljpp2c*#8-3 were transformed with empty vector and oe-LjMPK6 (T224A Y226F) (non-phosphorylated form of LjMPK6) via hairy root transformation. (**A**) Analysis of transcript abundance of *LjMPK6* in different hairy roots. Error bars represent SE of experimental values from three technical replicates. (**B**) Nodule numbers per plant at 3 weeks post inoculation (3 wpi) in transformed hairy roots. Error bars represent SE of ~13 plants analyzed for each genotype. ^**^p < 0.01, Student’s *t*-test. (**C**) Immunoblot analysis of protein level of overexpressed non-phosphorylatable LjMPK6 (anti-HA) and endogenous phosphorylated LjMPK6 (anti-p44/42) from those hairy roots. Equal loading of total protein was demonstrated by Coomassie Brilliant Blue (CBB) staining.

To further ascertain whether or not dephosphorylation of LjMPK6 affects nodule development, we constructed a non-phosphorylatable version of LjMPK6, LjMPK6^T224A; Y226F^ and introduced into wild-type MG20 or the *ljpp2c-KO* mutant by hairy root transformation (Figure 5A). Compared with the empty vector control, overexpression of LjMPK6^T224A; Y226F^ reduced the phosphorylation of MAPKs in Lotus plants, especially in the *ljpp2c-KO* mutant background (Figure 5C). Interestingly, compared to empty vector control, nodule numbers were significantly enhanced in LjMPK6^T224A; Y226F^ overexpression hairy roots of both MG20 and *ljpp2c*#8-3 plants, suggesting that LjMPK6 phosphorylation/dephosphorylation homeostasis indeed plays an important role for nodule organogenesis (Figure 5B).

## 3. Discussion

SymRK plays a key role in the NF signaling pathway during legume-rhizobia symbiotic interaction. SymRK-interacting protein SIP2 is required for early symbiotic signal transduction and MAPK signaling during nodule symbiosis [24]. As one of the phosphorylation substrates of SIP2, LjMPK6 is essential for nodulation [25]. However, downstream nodulation events and regulatory components of LjMPK6 signaling are largely unknown. Here, we demonstrated that LjPP2C dephosphorylates LjMPK6 in vitro and LjPP2C is required for MAPK dephosphorylation and nodule organogenesis in response to rhizobial inoculation.

Our phosphatase assay demonstrated that LjPP2C is a genuine protein phosphatase acting on the substrate LjMPK6, in accordance with its pivotal role in cellular signal transduction as reported for other plant PP2Cs in response to various environmental and developmental stimuli. For instance, a phosphatase 2C-1 (PP2C-1) allele from soybean dephosphorylates transcription factor GmBZR1 involved in brassinosteroid signaling [27]. Arabidopsis PP2C38 is an active phosphatase that negatively regulates cytoplasmic kinase BIK1-mediated immune signaling [28]. Rice XB15 harbors PP2C activity and negatively regulates the receptor kinase XA21-mediated innate immune response [29].

Only a few PP2C phosphatases have been identified in *Lotus japonicus*, largely due to the poorly annotated genome and their complicated biological functions. *LjNPP2C1*, encoding a Mg^2+^- or Mn^2+^-dependent PP2C, is specifically induced in *L. japonicus* nodules and functions at both early and late stages of nodule development, whereas the *LjPP2C2* gene was expressed at a similar level in nodules and roots [30]. In contrast, LjPP2C reported in this study appears to play an essential role in regulating nodule development, likely downstream of infection thread formation.

The *L. japonicus* 2C-type protein phosphatase LjPP2C interacts with the previously identified LjMPK6, which was shown to play an essential regulatory role in nodule development [25]. As a typical MAPK phosphatase, LjPP2C mainly functions through its dephosphorylation activity on LjMPK6. Compared to wild type MG20, a reduced nodule number and a higher level of LjMPK6 phosphorylation is observed in *ljpp2c* mutant plants, suggesting that a tight regulation of LjMPK6 phosphorylation is required for fine-tuning nodule organogenesis. In consideration of the immune signaling role of numerous plant kinase-PP2C phosphatase pairs previously reported [31], our work has extended our understanding of MAPK signaling in legume-rhizobia symbiosis and provides clues for further research on nodule developmental regulation mediated by protein phosphatases.

## 4. Materials and Methods

### 4.1. Plant Materials and Growth Conditions

*Lotus japonicus* seedlings were grown in pots containing perlite:vermiculite mixture (1:2, v/v) supplied with a 1/2 B&D nitrogen-free nutrient solution. *Nicotiana benthamiana* seedlings were grown in pots filled with perlite:vermiculite:nutrient soil mixture (1:1:1,v/v/v). All plants were cultivated in a growth chamber under a 16-h light/8-h dark cycle at 22 °C and 60% relative humidity.

### 4.2. Plasmid Construction

To create a *ljpp2c* mutant in *L. japonicus*, the web tool CRISPR-P 2.0 (http://cbi.hzau.edu.cn/crispr/) was used to design appropriate guide RNAs for a CRISPR/Cas9-mediated gene knockout approach. Two guide RNAs were cloned into one construct to improve gene-editing efficiency [26]. To create *LjPP2C* overexpression construct, full-length cDNA of *LjPP2C* was PCR-amplified from wild type *Lotus japonicus* MG20 genomic DNA and was inserted into pUB-Hyg vector [32]. All constructs were validated by DNA sequencing and were then transformed into *Agrobacterium tumefaciens* strain EHA105 for stable transformation into *L. japonicus* MG20 as described previously [33]. Transgenic plants were screened for hygromycin resistance and confirmed by PCR genotyping.

To create AD fusion constructs for pairwise yeast two-hybrid (Y2H) assay, full-length cDNA sequences of *LjMPK3*, *LjMPK4*, and *LjMPK6* were amplified by PCR and inserted into vector pGADT7 (Takara Bio, Beijing, China) to generate AD-LjMPK3, AD-LjMPK4, and AD-LjMPK6, respectively. The full-length cDNA sequence of *LjPP2C* and *LjMPK6* were amplified and inserted into pGBKT7 (Takara Bio) vector to generate BD-LjPP2C and BD-LjMPK6, respectively. All constructs were cloned using Phusion DNA polymerase (New England Biolabs, Ipswich, MA, USA) according to the one-step enzymatic assembly method protocol.

For promoter analysis, a ~3 kb DNA fragment of *LjPP2C* promoter was amplified using PCR and introduced into the pUB-Hyg plasmid to replace the Ubiquitin promoter and fused to tYFPnls (nuclear-localized triple-YFP) reporter [34].

To create plasmids for in vitro pull-down assay, full-length cDNA sequences of *LjMPK6*, *LjPP2C* were PCR amplified and cloned into pGEX-6p-1 and pMAL-c2x, respectively, to obtain the corresponding GST-tagged and MBP-tagged fusion proteins. To create overexpression vector for hairy root transformation in *L. japonicus*, 3*HA tagged full-length cDNA sequence of *LjMPK6* with two introduced point mutations (T224A and Y226F) was cloned into the empty vector pUB-GFP [32]. Sequences of corresponding primers used for plasmids construction are listed in Table S1.

### 4.3. Yeast Two-Hybrid Assay

Yeast two-hybrid (Y2H) screening was performed according to the manufacturer’s instruction (Takara Bio, Beijing, China), using *Saccharomyces cerevisiae* Y187 strain harboring the BD-LjMPK6 fusion construct as bait and *S. cerevisiae* AH109 strain harboring AD fusion construct of Lotus cDNA library derived from root and nodule tissues as prey. A total of 1 × 10^7^ transformants were screened, and positive colonies growing on SD/-Leu-Trp-His-Ade (SD/-4) medium were selected for plasmid extraction and sequence analysis. The candidates were retransformed into yeast for pairwise Y2H assay to validate the interaction results. Cells of yeast strain Y187 transformed with AD fusion constructs and cells of yeast strain AH109 transformed with the BD fusion construct were respectively spreaded onto SD/-Leu or SD/-Trp agar plates using a LiAc-mediated yeast transformation protocol and then mated overnight in 2×YPDA. Aliquots (10 μL) of diploid yeast cells were spotted onto SD/-Leu-Trp (SD/-2) and SD/-Leu-Trp-His-Ade (SD/-4) medium to verify protein-protein interactions. Interactions between p53 (or lamin, Lam) and SV40 were used as positive or negative controls, respectively. Yeast growth was monitored for up to 5 days at 30 °C.

### 4.4. In Vitro Pull-Down Assay

Protein expression of GST-tagged LjMPK6 and MBP-tagged LjPP2C was induced in *Escherichia coli* strain BL21 (DE3) by 0.1~0.5 mM isopropylthio-β-galactoside (IPTG) for 4 h at 30 °C and purified with glutathione resin (GenScript, Nanjing, China) and Amylose resin (New England Biolabs), respectively. Purified proteins were then incubated with glutathione resin in PBS buffer (2 mM KH_2_PO_4_, 8 mM Na_2_HPO_4_, 136 mM NaCl, and 2.6 mM KCl, pH7.4) for 30 min at 4 °C and separated by centrifugation. The supernatant was discarded, and the glutathione resin was washed at least three times with PBS buffer. Proteins retained with the glutathione resin were boiled in SDS loading buffer (50 mM Tris-HCl, pH 6.8, 2% SDS (w/v), 0.1% bromophenol blue, 10% glycerol, 1% β-mercaptoethanol) and separated by SDS-PAGE electrophoresis. Corresponding antibodies against GST or MBP (PhytoAB, San Jose, CA, USA) were used for immunoblot analysis.

### 4.5. Phosphatase Assay

The kinase assay between SIP2 and LjMPK6 was performed in advance to prepare phosphorylated GST-tagged LjMPK6, which was then purified by glutathione resin. The in vitro phosphatase assay of LjPP2C was performed by incubating phosphorylated LjMPK6 with LjPP2C in a buffer containing 50 mM Tris-HCl, pH 7.5, 10 mM MgCl_2_, 0.1% Triton-X100, and 1 mM DTT at 30 °C for 30 min. Reactions were stopped by adding 2×SDS loading buffer and boiling for 5 minutes. The remaining phosphorylated LjMPK6 was identified by immunoblotting with anti-phospho-p44/42 (pThr-X-pTyr) MAPK antibody (Cell Signaling Technology, Danvers, MA, USA). 

To detect the phosphatase activity of LjPP2C on LjMPK6 in *L. japonicus* in vivo, roots of MG20 and ljpp2c#8-3 were transformed with empty vector or oe-LjMPK6 (T224A Y226F) (non-phosphorylatable form of LjMPK6). Transgenic hairy roots of above materials were grounded into fine powder in liquid nitrogen. Total protein was extracted using sample buffer containing 50 mM Tris-HCl, pH 6.8, 5% (w/v) SDS, 10 mM DTT, 100 μM PMSF (phenylmethylsulfonyl fluoride), and 1 mM sodium pyrophosphate. Phosphorylated LjMPK6 was identified by immunoblotting with anti-phospho-p44/42 (pThr-X-pTyr) MAPK antibody.

### 4.6. RNA Extraction and Reverse-Transcription Quantitative PCR (qRT-PCR)

Plant total RNA was isolated using TRIzol reagent (Thermo Fisher Scientific, Hampton, NH, USA) and treated with DNase (Promega, Madison, WI, USA) to eliminate genomic DNA contamination. Less than 1 μg total RNA was used to synthesize first-strand cDNA using oligo(dT) primer according to the instructions of the cDNA synthesis kit (TransGen Biotech, Beijing, China). RT-qPCR was performed on a Roche Light Cycler (thermal cycle: 95 °C for 10 s, 40 cycles of 95 °C for 5 s and 60 °C for 30 s, followed by a melting curve stage at 95 °C for 15 s and a temperature gradient from 65 °C to 95 °C at a rate of 1 °C s^−1^) based on the instruction of the one-step SYBR Prime Script RT-PCR Kit II (Takara Bio). The housekeeping genes *LjATPase* (AW719841) or *LjUBQ* served as reference genes for relative fold expression changes using the 2^−△△Ct^ method. All reactions were performed with three technical replicates.

### 4.7. Hairy Root Transformation and Nodulation Assays

*L. japonicus* hairy roots were obtained by transformation with *Agrobacterium rhizogenes* LBA1334 containing the overexpression construct pUB-GFP-LjUBQ1pro::LjMPK6(T224A;Y226F)-3*HA as described previously [35]. Plants transformed with the empty vector (EV) pUB-GFP-3*HA were used as control. Regenerated hairy roots were screened for GFP (green fluorescent protein) fluorescence. Only one GFP-positive root was retained for each plant, and all other roots were removed.

One-week-old seedlings were transferred from Petri dishes to pots containing perlite:vermiculite (1:2, v/v). Plants were watered with 1/2 B&D nitrogen-free nutrient solution for 1 week and were then inoculated with 1 mL of *Mesorhizobium loti* MAFF303099 (OD600=0.1) expressing the red fluorescent marker mCherry (pQDN03-ptrp::mCherry) [36,37]. At 3 weeks post inoculation (3 wpi), roots were collected for counting nodules, and root samples were harvested for protein immunoblot analysis.

### 4.8. Accession Numbers

Sequence data for the following proteins can be found in the Lotus database (v3.0, https://lotus.au.dk). *LjPP2C*, Lj2g3v2292680.1; *LjMPK3*, Lj3g3v3087330.1; *LjMPK4*, Lj4g3v2989020.1; *LjMPK6*, Lj4g3v0510090.1; *SIP2*, Lj3g3v2040150.1.

## Supplementary Materials

Supplementary materials can be found at https://www.mdpi.com/1422-0067/21/15/5565/s1.

## Abbreviations

CRISPR	clustered regularly interspaced short palindromic repeats
GFP	Green Fluorescent Protein
GST	Glutathione S-transferase
MAPK	mitogen-activated protein kinase
MBP	maltose-binding protein
NF	nod factor
NIN	nodule inception gene
PP2C	type 2C protein phosphatase
RLK	receptor-like kinase
Y2H	yeast two-hybrid
