# Supplementary material for: Dephosphorylation of LjMPK6 by Phosphatase LjPP2C is Involved in Regulating Nodule Organogenesis in Lotus japonicus
Source: Int J Mol Sci. 2020 Aug 3;21(15):5565. doi: 10.3390/ijms21155565 (PMC7432216; doi:10.3390/ijms21155565)
Supplement: Supplementary file 1 [file ijms-21-05565-s001.pdf]

Supplemental Table 1. Primers used in this study.

Primers used for cloning into protein expression vectors in yeast

	Forward	Reverse
Cloning into pGADT7		
LjMPK6 (Lj4g3v0510090.1)	5'-GGCATCGATACGGGATCCATATGGAAGGAGGAGGAGGTGC-3'	5'-CATCTGCAGCTCGAGCTCCTAATGCTGTTCAAAATCAAAGCTG-3'
Cloning into pGBKT7		
LjPP2C (Lj2g3v2292680.1)	5'-CCGAATTCCCGGGGATCCGTATGTCTTGCTCCGTCG-3'	5'-GTTATGCGGCCGCTGCAGTTAAATATAGTGCTCC-3'

Primers used for cloning into protein expression vectors in *E.coli*

	Forward	Reverse
Cloning into pMALc2x		
LjPP2C (Lj2g3v2292680.1)	5'-ATTTCAGAATTCGGATCC ATGTCTTGCTCCGTCGCAGT-3'	5'-TGCCAAGCTTGCCTGCAG TTAAATATAGTGCTCCAATTTGATG-3'
Cloning into pGEX-6P-1		
LjSIP2 (Lj3g3v2040150.1)	5'-CAGGGGCCCCTGGGATCC ATGAGGCCGATTCAACTAC-3'	5'-GTCAGTCACGATGCGGCCGC CTATGAAGAAAGAGACCTGG-3'
LjMPK6 (Lj4g3v0510090.1)	5'-CAGGGGCCCCTGGGATCC ATGTCTTGCTCCGTCG-3'	5'-GTCAGTCACGATGCGGCCGC TTAAATATAGTGCTCCA-3'
Cloning into pET-28a		
LjSIP2 (Lj3g3v2040150.1)	5'- TGGTGCTCGAGTGCGGCCGC ATGAGGCCGATTCAACTAC-3'	5'- CAAATGGGTCGCGGATCC CTATGAAGAAAGAGACCTGG-3'
LjMPK6 (Lj4g3v0510090.1)	5'-TGGTGCTCGAGTGCGGCCGC CTGCTGATACTCAGGGTTA-3'	5'-CAAATGGGTCGCGGATCC ATGGAAGGAGGAGGAGGT-3'

Primers used for cloning into vectors for protein expression in *N. benthamiana* leaves or *L. japonicus* hairy roots

	Forward	Reverse
Cloning into pUB-GFP-3×FLAG (plus 6×His tag)		
LjPP2C (Lj2g3v2292680.1)	5'-GTCCTTATAGTCGGTACC (GTGGTGGTGGTGGTGGTG) AATATAGTGCTCCAATTTGATG-3'	5'-ATGTGATTACAGTCTAGA ATGTCTTGCTCCGTCGCA-3'
LjMPK6 (Lj4g3v0510090.1)	5'-GTCCTTATAGTCGGTACC (GTGGTGGTGGTGGTGGTG) CTGCTGATACTCAGGGTT-3'	5'-ATGTGATTACAGTCTAGA ATGGAAGGAGGAGGAGGT-3'
Cloning into pUB-GFP-3×HA		
LjMPK6 (Lj4g3v0510090.1)	5'-ATCGTATGGGTAGGTACC CTGCTGATACTCAGGGTT-3'	5'-ATGTGATTACAGTCTAGA ATGGAAGGAGGAGGAGGT-3'
Cloning into p1300-35S-sGFP-HA		
LjPP2C (Lj2g3v2292680.1)	5'-CGCGCCACTAGTGGATCC ATGTCTTGCTCCGTCG-3'	5'-GCCCTTGCTCACCCCGGG AATATAGTGCTCCAATT-3'
Cloning into p1300-35S-mCherry-FLAG		
LjMPK6 (Lj4g3v0510090.1)	5'-GGTACCCTCGAGGTCGAC CTGCTGATACTCAGGGTT-3'	5'-GCCTGGCGCGCCACTAGT ATGGAAGGAGGAGGAGGT-3'

Primers used for introducing mutated versions of proteins

	Forward	Reverse
LjPP2C (Lj2g3v2292680.1)		
D169N; D330N	5'-CTCCATGCCCATTAAATACGCCA-3'<br>5'-CCCATAATCCATTTGAAGCTAAT-3'	5'-TGGCGTATTTAATGGGCATGGAG-3'<br>5'-ATTAGCTTCAAATGGATTATGGG-3'
LjMPK6 (Lj4g3v0510090.1)		
T224A Y226F	5'-CTGATTTTATGGCTGAA<br>TTTGTTGTCACAAG-3'	5'-CTTGTGACAACAAATTC<br>AGCCATAAAATCAG-3'

Primers used for cloning promoter

LjPP2C promoter	
forward	5'-ACTGACCACCCGGGGAGTTGTTGCTTGTTCTTGCTC-3'
reverse	5'-ATCTACAGCGCTGAGATGTTATTGTTTGTTTGAGTCAA-3'

Primers used for qRT-PCR

	Forward	Reverse
LjATPase	5'-CAATGTCGCCAAGGCCCATGGTG-3'	5'-AACACCACTCTCGATCATTTCTCTG-3'
LjPP2C	5'-GATTGCCGTGCTGTCATTAG-3'	5'-AATCAACATAGCCACCCAGA-3'
